# Record of Medical Science

**Published:** 1856-09

**Authors:** 


From the Medical Examiner.

*Record of Medical Science.*

*Purification of Water supplied to Towns, etc.*—At a recent meeting of of the Society of Arts, the method proposed by Dr. Clark for purifying water for the supply of towns was described by him, and its applicability for this purpose discussed.

The substances with which water is contaminated may be in two states —suspended and dissolved; both may contain mineral and organic substance.

Spring water contains from 1-200 to 1-1000, or even 2-1000, dissolved substance, but no suspended substance. This is the case with many kinds of water in and around London; but, when collected at the surface in reservoirs, and exposed to light and air, vegetation commences, and is succeeded by the development of animalcules. After a time, both the plant and animal organisms pass into a state of putrefaction, and become a source of serious contamination.

The water of rivers generally contains less dissolved substance than that of the springs in the same district, and it also contains suspended substances of various kinds that are washed into the rivers from the banks by small streams, rivers, &c.

The separation of suspended substance is effected either by subsidence or by filtration.

The nature of the dissolved substance depends upon the kinds of strata traversed by the water; it generally consists, for the most part, of calcareous salts—sometimes with magnesian salts—alkaline salts, ammoniacal salts, rarely, and in small amounts.

The calcareous and magnesian salts communicate to water the character of *hardness.* This character varies considerably in amount in different kinds of water, and is expressed in degrees, each degree of hardness being as much hardness as a grain of chalk, or the lime, or the calcium, in a grain of chalk, would produce in a gallon of water, by whatever means it may be dissolved.

The hardness of most of the water around London is owing to the presence of dissolved carbonate of lime. The amount is so large, that the average supply of water to a single family would yeild in eight months 100 pounds of chalk, and in 100 gallons of water there is enough to destroy 35 ounces of soap.

Carbonate of lime itself is very sparingly soluble in water; probably 5000 gallons would be requisite to dissolve one pound avoirdupois. But when combined with an additional amount of carbonic acid, it forms bicarbonate of lime, which is so much more soluble in water, that one pound of carbonate with seven ounces additional of carbonic acid would dissolve in 400 gallons of water; and this is about the amount present in well-water from the chalk strata.

The carbonic acid may be separated from carbonate of lime by heating, as in the ordinary operation of lime-burning, and the lime thus obtained is still more soluble in water than the bicarbonate of lime; so that a pound of carbonate of lime, consisting of—

Lime.................................9 ounces,
Carbonic Acid,.........................7 ounces, yields a quantity of lime that may be dissolved in 40 gallons of water.

Thus it appears that carbonate of lime, itself scarcely at all soluble in water, may be rendered soluble in two different ways—either by being deprived of its carbonic acid, or by combining with an additional quantity of carbonic acid.

It is by the latter of these two changes that water, in traversing chalk strata becomes so highly impregnated with carbonate of lime; for carbonic acid is always abstracted from the atmosphere by water during its condensation as rain, &c., and a further amount is frequently dissolved by the water in percolating the vegetable soil.

To separate this dissolved carbonate of lime, so far as may be practicable, is the object of Dr. Clark's method of purification. It is based upon the fact that when a solution of bicarbonate of lime, such as ordinary water, is mixed with a solution of lime, half the carbonic acid is abstracted from the bicarbonate, and both lime and bicarbonate of lime are converted into the very sparingly soluble carbonate.

When this operation is so managed that the lime added is just sufficient to form carbonate with the surplus carbonic acid in the bicarbonate, almost the whole of the dissolved carbonate will be removed from the water, and only so much will remain dissolved as corresponds with the solubility of carbonate of lime.

| | | | |
|---|---|---|---|
| Bicarbonate of lime in 400 gallons. | Carbonate of lime 16 oz. | | =2 lbs. |
| | Carbonic acid...... 7 oz. | =16 oz. carb. of lime | |
| Lime in 40 gallons of water. | ........................ 9 oz. | | |

This residual carbonate of lime is always small in amount. Supposing in the above instance, the 440 gallons contained 1½ oz. dissolved carbonate of lime, 1011ths, or 16 oz. would be separated, and only 17½ oz. be left in solution. The water, before being softened, would destroy 35 oz. of soap for every 100 gallons; after being softened, the same quantity would destroy only 5 oz.

Most water contains, besides carbonate of lime, calcareous and magnesian sulphates, clorides, &c. These substances communicate hardness to water, as well as carbonate of lime; but there is this difference—that the hardness, owing to the presence of these substances, is not removed by liming. This, however, is not of any practical importance, so far as regards the purification of the water supply of London by this method; for the hardness of the water around London is chiefly owing to carbonate of lime.

Without, perhaps, being prejudicial to health, the disadvantages arising from the presence of carbonate of lime in water, are numerous and considerable.

1. It is the principal cause of the incrustation of steam-engine boilers.
2. It causes a great, and at the same time useless, increase in the consumption of soap, and is deposited in dirty linen in such a manner as to fix the dirt, and prevent its being rendered white.

3. For many culinary purposes it is less suitable than soft water.

Dr. Clark's method is remarkable, inasmuch as it differs from most chemical operations in not introducing any other substance into the water in place of the carbonate of lime separated; and moreover, the separation is effected without the use of any substance foreign to the water in its natural state.

There is another effect produced by this method of purifying water, which does not appear to have been at first anticipated by Dr. Clark. It is the removal of organic substance.

In general the wholesomeness of water is much more affected by the presence of organic substance than by mineral substance; and it seems to be a fact well established by observation, that some of the poisons producing epidemic disease find a congenial habitat in water contaminated with organic substance. Moreover, when organic substance in water undergoes putrefaction, the sulphates always present in water are decomposed, and sulphurated hydrogen generated. The deleterious character of the water of the Niger was ascribed by the late Professor Daniell to this circumstance.

The amount of organic substance in water may be very minute, but it must not on that account be regarded as insignificant. The amount of organic substance in the most defective kinds of water supplied in London, is very small in proportion to the mineral substance; but it is generally considered by recognized authorities, that, under certain conditions, this organic substance may acquire such a state as to produce disease in those drinking it habitually. In this respect the cause of disease existing in water is analogous to that known as sausage-poison, and that producing the frequent fatal effects of a cut with a dissecting knife, neither of which appear to be chemically tangible.

Investigations relating to the last epidemic of cholera have shown that in one district in London, containing a population of 500,000, which were chiefly supplied with water by two different companies, there were over 4,000 deaths from cholera during the epidemic. The only recognizable difference in the conditions and modes of life of the inhabitants, was, that one portion were supplied with water of good quality, drawn frown a point high up the Thames; while the other portion were supplied with water drawn from a lower point of the river, where it was profusely contaminated with town-drainage. It proved, upon inquiry, that the mortality among the former portion was 37 in 10,000, while among the other portion it was 130 in 10,000, or three-and-a-half times as great as in those houses supplied with the better water. Further inquiry showed that in the epidemic of 1848–49 the mortality was uniform throughout the district. There was no such difference between the houses supplied with water by the two companies, the mortality being in one case 118, and in the other 125; but at that period both companies drew their water from nearly the same part of the Thames, low down, where it was contaminated with town-drainage.

The method of purification proposed by Dr. Clark not only effects the separation of carbonate of lime, which as regards the wholesomeness of water is of secondary importance, but it also separates organic substance. At the print works, in Manchester, it is applied specially for this purpose, and in an experiment made upon 3,000,000 gallons at the Chelsea Water Works it is stated by Dr. Miller that the amount of organic substance was reduced to one-third.

Some doubt was expressed by speakers who took part in the discussion as to whether the organic substance removed by liming was that suspended or that in solution. Both are in fact removed, but it does not appear that there are any grounds for regarding the one more prejudicial to health than the other.

The removal from water of the carbonate of lime dissolved by carbonic acid, has also, indirectly, an influence upon the contamination with organic substance, by serving as a preventive of vegetation, and of the consequent development of animal organism.

When chalk spring water is pumped up from a well and exposed to light and air, in a clean glass vessel, capable of holding a few gallons, with a glass covering, and so exposed that the changes can be observed as they take place from day to day, it will be seen that all around the sides and bottom a green vegetation will appear in summer time within a few days. In process of time this vegetation tends to a brown, and if a close observation be made, a slight incrustation may be discovered, partly to float on the surface of the water, and partly to adhere to the sides and bottom of the vessel. This incrustation consists of carbonate of lime, slowly precipitated from the water by the separation of the duplicate dose of carbonic acid that kept the carbonate of lime dissolved. It is this carbonic acid that serves as the food of plants, furnishing carbon to them, and the carbonate of lime that was kept in solution by it forms the mineral part of the incrustation. If the glass vessel, after having been exposed as described for several weeks, be emptied, a dirty brownish incrustation, including vegetable substance, may be very well seen, all down the sides, and on the bottom. This brownish incrustation has a strong, offensive, marshy smell. If side by side with the spring water there be exposed, in a similar glass vessel, the same water, previously softened, the softened water will continue for weeks and months unaltered, while that unsoftened water is becoming more and more contaminated by vegetation.

So long back as 1851, the commissioners appointed to report on the quality of the water supplied to London, remarked, that "it appeared to be only a question of time, when the sense of the violation of the river purity (by town drainage) would decide the public mind to the entire abandonment of the Thames as a source of supply, unless artificial means of purification were devised and applied." They also stated, "that a careful series of experiments left no doubt in thei minds that the means of conducting this process are certain in their results, and sufficiently simple to be left to the execution of a workman of ordinary intelligence, that the process falls easily into the routine operations of water-works * * is not attended with any peculiar difficulty on the large scale, and that the softening of Thames water in its ordinary condition by this process is perfectly practicable, at a cost which would, on the average, increase the price charged to the consumer only four per cent."

Nevertheless, there is only one instance in which this process has been applied to the purification of water supplied for general purposes. At the Plumstead Water-Works, near Woolwich, it has been in successful operation for the last year and a half. The water supplied by this Company is derived from the chalk by boring, and has about twenty degrees hardness, which is reduced to eight degrees by liming. The works are capable of supplying 600,000 gallons daily, and at the present time about 3,000 houses are supplied.

Eight months after the Plumstead Water Company had been carrying on the softening process with success, and much to the satisfaction of the consumers, it occurred to the company to try how far the consumers would continue to be satisfied with the water, if the softening process were omitted.

The consequence was, that by the twelfth day the surface of the unsoftened water in the reservoirs, though daily renewed, was covered with masses of confervæ to such an extent, that scarce a square inch could be found clear, and a powerful stench of decaying vegetable substance was evolved. Complaints of the water soon followed, and the experiment was discontinued.

In the course of the discussion, Mr. Braithwaite put forward objections to the application of Dr. Clark's method of purification, on the ground that a certain amount of lime was necessary for maintaing the functions of animal life, and cited, in support of his argument, experiments made by Liebig, upon pigeons and cows. But, the quantity of lime supplied in solid food is much more than adequate to these requirements; in many districts, the water consumed by large populations, and by great numbers of cattle, is soft with a very small amount of lime in any state; and further, the lime salt, required for the formation of bone, is not carbonate, but phosphate of lime, which is never present in water to more than an infinitesimal amount. Moreover, the experiments cited by Mr. Braithwaite are quite inapplicable to the case in question, because, in those experiments, lime was entirely abstracted from the solid food, as well as from the water supplied to the animals.—*London Pharmaceutical Journal.*

---

*On the Pathology of Hooping Cough.*—After enumerating some of the many discrepant and imaginative theories of the nature of hooping cough that writers on the subject had indulged in, the author stated, in answer to the question, To what category of derangements do the most constant and characteristic features of the disease the most intimately unite it?—that, in his opinion, it was to the contagious fevers—to those diseases which consist of the assumption into the body of some specific *materies morbi* introduced from without, and undergoing a certain process of self-multiplication within the system—to the zymotic diseases; in fact, in favor of this view, he said, there was this threefold evidence:

1st.—That hooping cough was contagious.

2d.—That it runs a definite course, having certain premonitory signs: certain phenomena when the disease has attained its height, and certain sequelæ.

3d. That it is self-prophylactic; a person having had it once, does not have it again.

Now the three circumstances—contagion, definite course, and self-prophylaxis, are, he maintained, *par excellence*, the three characteristic circumstances of the contagious fevers, and the possession by any disease of these three features would always be, to him, a sufficient warrant for its admission into that family of disorders. The author then thus stated, in more exact terms, his views: That the catching of hooping cough depends upon the inoculation of the system with a specific poison; that this poison chooses for itself a certain eliminatory surface as its emunctory; that the surface that it so chooses is the respiratory tract of the mucous membrane, from the conjunctiva to the ultimate bronchial tubes, although the whole of the tract need not be involved in every case; that its material presence gives rise to an exalted sensibility and inflammation of the part; and that the exalted sensibility and inflammation constitute the proximate cause of the specific symptoms. The author's conviction of the correctness of the above theory was based on the following considerations:

*a.* The premonitory symptoms of catarrh, injection of the eyes, coryza, &c.

*b.* The symptoms of vascular disturbance of the trachea, bronchial tubes, large and small, down even, in many cases, to the ultimate lung structure, that generally accompany or follow the cough.

*c.* The intermediate position in regard to time, of the laryngeal, between the nasal and the bronchial symptoms, implying a creeping down of the condition of the mucous membrane in a regular course.

*d.* The power which one child will have, who does not hoop, of communicating the disease to another who will; showing that the spasmodic part of the affection is non-essential.

*e.* The eliminatory power of the surface, which is consistent with the supposed final cause of its being affected.

*f.* The support derived from the whole weight of the argument of analogy.

Dr. Salter finished his paper by refuting, in succession, certain objections to his theory, which he could conceive others to make, but which, from our limited space, we are unable to enumerate.

Dr. Richardson believed that hooping cough belonged to the zymotic class of diseases. He advised, that Dr. Salter should ascertain if the mucous is inoculable, and suggested the pig as a fit animal. He had seen pigs with croup, small-pox, measles and plague. Inoculation acts well in modifying disease, by introducing but a very small dose at once, and for the same purpose, it is advantageous to inoculate from matter obtained from animals which had been the subjects of the disease.

Dr. Edward Smith had proved, in a paper published in the "Transactions" of the Royal Medical and Chirurgical Society, that the deaths from hooping-cough were mainly due to bronchitis; but he believed that inflammation was only an accident, and not an essence of the disease. He had doubts as to its being a blood disease, in the sense of being introduced into the system in the form of an organic poison; but, at all events, he considered that the spasm is all that distinguishes it necessarily from a common cough. The secretion is in great part due to the violent spasmodic cough; and the plan of treatment, which in a large experience he had found suitable, was to arrest the spasm, and thereby both the cough and the secretion; so that, in a very short time, the attack is reduced to the condition of a common cough. Since the disease may thus be cut short in probably all uncomplicated cases, and yet not be more liab e to return than when allowed to run its course, he could not support the author's theor*y* of elimination of the poison in the secretion of the mu cous membrane of the larynx and bronchi. It is, however, just possible that the supposition of the gradual destruction in the system of the poison might account for the non-recurrence of the disease when thus cut short; but that would be an assumption, and, if true, would render the theory of elimination of no value in practice. He strongly commended the employment of small and increasing doses of morphia, on the plan laid down by him in a paper published in the *Edinburgh Medical Journal* for May.

Dr. Wynn believed that the disease does not run through a regular course. He admitted that it is a contagious disease, but its evidences are mainly nervous.

Dr. Webster remarked upon the difference of opinion existing as to the pathology of the disease. He did not consider it a contagious disease in the sense that measles is contagious, and he did not think it ran through a definite course. It may also recur. It is more common in the winter, and with northerly winds and frosty weather. Change of wind and air are often beneficial. Treatment will often cut short the attack. He believed the disease chiefly affected the head. It is more fatal amongst female young children.

Dr. Camps thought that the author's cases must have been complicated with some inflammatory condition. Mild temperature is beneficial. It does not run a definite course, and treatment may cut it short.

Dr. Radcliffe did not believe in the necessary connection between hooping-cough and true inflammation, and when that complication exists, the hoop is suspended. The disease is capable of being arrested, and hence does not run through a definite course.

Dr. Headland did not agree with the peripheral theory, and thought that the centric theory accounted for the production of the paroxysm. Many poisons do not act upon eliminating parts of the system. He did not approve the experimentum crusis.

The author replied.—*Proceedings of the Medical Society of London, in London Lancet.*

---

*On the Dropsy of Pregnancy.* By M. Becquerel.

Four forms of dropsy are observed in pregnant women, which are far from being of the same importance.

1. *Mechanical Dropsies*, perhaps the most common, are due to the pressure exerted by the gravid uterus, their production being favored by the lesser density of the blood in pregnant women, and the slight diminution of albumen that exists in its serum. These dropsies are confined to the lower extremities, are of no importance beyond their inconvenience, and disappear after delivery.

2. *Dropsies due to Changes in the Blood, but unaccompanied by Albuminuria.*—The change in the blood which induces these dropsies, consists in a diminution in the amount of the albumen of the serum, a diminution that is sometimes considerable, and for which we can assign no other cause than the fact of the pregnancy, and its influence on the various immediate principles of the blood. This description of dropsy, like the two next, tends to become general. It is of importance to distinguish it from the two others, and especially the 4th, for it does not predispose to eclampsia. It is by analysis of the blood alone that we can establish its existence. It disappears also after pregnancy, but far more slowly. It has been observed that women suffering from it remain feeble for a long period, their "getting up" being slow and difficult.

3. *Dropsies with Changes in the Blood and Albuminuria, but without*

*Bright's Disease, properly so called.*—These dropsies are the consequence of the diminution of the albumen of the blood, produced by its deperdition through the kidney. Until lately it was supposed that such loss might take place without material lesion of the kidney; but from the investigations made by M. Robin and the author, it results that this albuminuria is due to a special modification taking place in the epithelial cells of the tubuli, a modification consisting in the infiltration of the cells and tubuli by numerous granules of a proteric nature. This infiltration is an[illegible]logous to that which M. Robin had already found in choleraic albuminuria, and like it is susceptible of cure. The absolute diagnosis during life of this disease from Bright's affection is very difficult, and yet it is highly important, as the prognosis must be entirely based upon it. It is in women who are the subjects of these dropsies that we have to fear eclampsia, and the predisposition to puerperal peritonitis. Eclampsia, is not, however, a necessary consequence; and when we find general dropsy, change in the blood, and albuminuria co-existing, we still cannot affirm that this terrible accident will follow. On the other hand, whenever we find eclampsia we are certain of finding, not only dropsy, but albuminous urine, and change in the blood. In respect to the termination of this form of dropsy it may be observed, that if eclampsia does not supervene, a cure is almost certain, while, in the case of its occurring the result is dependent upon that of the eclampsia.

4. *Dropsies due to Bright's Disease.*—It is very important to establish the diagnosis of this form. We may lay stress upon the somewhat larger quantity of albumen, the presence of fragments of the tubuli, of fibrinous filaments, and fatty globules. When eclampsia complicates this form it is invariably fatal; and even when eclampsia does not occ[illegible], the disease is not arrested after delivery. The dropsy continues to increase, the termination proving, after a certion period, fatal.—*London Medical Times, from Rev. Medico-Chirurgicale.*

---

*On the Treatment of Hæmoptysis.* By M. Aran.—M. Aran agrees with those who entirely condemn the employment of blood-letting in the treatment of hæmoptysis, as it only temporarily arrests the bleeding, while it is dangeous, owing to the debility, and increased susceptibility to the intercurrent affections it gives rise to. He has, for some time past, been engaged in testing the efficacy of the various hæmostatic agents employed in hæmoptysis; and in this paper he gives the results of his observations. He considers the essence of turpentine a most valuable remedy, given in d[illegible]ses of from 10 to 30 drops every hour, either in a spoonful of water, or mixed up with magnesia, as a bolus. Marked amendment usually occurs in a few hours, and in from twenty-four to thirty-six hours the bleeding ceases. It is less suitable for young or plethoric subjects with febrile action, than in weak cachectic individuals, exhibiting a tonic characteristics. Ergot of rye and ergotine are far less efficacious; but chloride of sodium, given in doses of 1 to 2½ drachms, proves very efficacious in some cases, and has the advantage of being always at hand. Among the astringents, tannin, and especially gallic acid, are to be recommended; the latter, while quite as efficacious, does not exert the same dessicating effect upon the tissues, or induce the obstinate constipation produced by tannin. As a mean dose, M. Aran gives 15 centigrammes (a centigramme is 1-7th of a grain) every hour or alternative hour. He has had little experience in the use of emetic and nauseating remedies; but in three cases in which veratrine was employed, the bleeding ceased as if by enchantment. This class of remedies, indeed, would deserve to stand in the first class of hæmostatic agents, were there not others possessing like efficacy, and yet not giving rise to the painful nausea these produce. M. Aran has derived great advantage from the combined use of digitalis and nitre. In ordinary cases, he gives in the twenty-four hours, 30 centigrammes of digitalis, and $1\frac{1}{2}$ gramme (a gramme is 15 grains) of nitre, divided into four doses; but in very severe cases, these doses may be very much increased, so that the digitalis has been given to the extent of $1\frac{1}{2}$ gramme, and the nitre to four grammes, without injuriously affecting the action of the heart, while the effect produced on the hemorrhage has been remarkable. Its arrest never, however, takes place so suddenly, under the use of these medicines, as when turpentine or gallic acid is employed.

In abundant, but not immediately dangerous hemorrhage, we can choose among any of the above-mentioned means. In extremely abundant hemorrhage, we must arrest the flow as speedily as possible, by agents which do not depress the powers of the economy too much, and which are not too slow in their operation. Neither ergot, acetate of lead, nor alum, is sufficient to meet the danger. Turpentine, gallic acid, chloride of sodium, or nitre with digitalis, can alone be trusted; but the necessity of increasing the dose, with the intensity of the hemorrhage, may perhaps render the chloride of sodium, and especially the nitre and digitalis, dangerous, through the possibility of the production of a too great depression of the heart's action. It is, therefore, to gallic acid, or turpentine, that we must chiefly trust in these severe cases; and we must not limit ourselves to their employment, but also endeavor to procure a temporary arrest of the hemorrhage by ligatures to the limbs, and the application of ice to the chest, allowing the means employed internally to consolidate this temporary cure.—*Med. Times and Gaz.*, *Jan.*, 1856, *from Gaz. Hop.*, 1855.

